# Actein induces autophagy and apoptosis in human bladder cancer by potentiating ROS/JNK and inhibiting AKT pathways

**DOI:** 10.18632/oncotarget.22274

**Published:** 2017-11-01

**Authors:** Lu Ji, Bing Zhong, Xi Jiang, Fei Mao, Gang Liu, Bin Song, Cheng-Yuan Wang, Yong Jiao, Jiang-Ping Wang, Zhi-Bin Xu, Xing Li, Bo Zhan

**Affiliations:** ^1^ Department of Urology, Huai’an First People's Hospital, Nanjing Medical University, Huai’an 223300, China; ^2^ Department of Orthopaedics, Huai’an First People's Hospital, Nanjing Medical University, Huai’an 223300, China; ^3^ Branch of Raw Material and Natural Products, Far East Biological Products Co. LTD., Nanjing 210009, China

**Keywords:** human bladder cancer, Actein (ACT), autophagy and apoptosis, ROS and JNK, AKT

## Abstract

Human bladder cancer is a common genitourinary malignant cancer worldwide. However, new therapeutic strategies are required to overcome its stagnated survival rate. Triterpene glycoside Actein (ACT), extracted from the herb black cohosh, suppresses the growth of human breast cancer cells. Our study attempted to explore the role of ACT in human bladder cancer cell growth and to reveal the underlying molecular mechanisms. We found that ACT significantly impeded the bladder cancer cell proliferation via induction of G2/M cycle arrest. Additionally, ACT administration triggered autophagy and apoptosis in bladder cancer cells, proved by the autophagosome formation, LC3B-II accumulation, improved cleavage of Caspases/poly (ADP-ribose) polymerase (PARP). Furthermore, reduction of reactive oxygen species (ROS) and p-c-Jun N-terminal kinase (JNK) could markedly reverse ACT-induced autophagy and apoptosis. In contrast, AKT and mammalian target of rapamycin (mTOR) were greatly de-phosphorylated by ACT, while suppressing AKT and mTOR activity could enhance the effects of ACT on apoptosis and autophagy induction. *In vivo*, ACT reduced the tumor growth with little toxicity. Taken together, our findings indicated that ACT suppressed cell proliferation, induced autophagy and apoptosis through promoting ROS/JNK activation, and blunting AKT pathway in human bladder cancer, which indicated that ACT might be an effective candidate against human bladder cancer in future.

## INTRODUCTION

Human bladder cancer is one of the most common genitourinary malignant cancers, leading to health issue worldwide [1, 2]. Presently, the existed therapeutic strategies, including radical cystectomy and chemotherapy, are far from to be satisfied considering the metastases and recurrence of human bladder cancer [3–5]. Thus, although advances have been made in radiotherapy, perioperative chemotherapy, and surgical technology, the 10-year survival rate remains grave after radical cystectomy [3, 6]. Hence, it is still urgently to find and develop new and effective strategies against bladder cancer.

The purified triterpene glycoside actein (β-D-xylopyranoside, ACT), isolated from black cohosh, was suggested to be effective against human breast cancer cells [7, 8]. Black cohosh was applied by the Native Americans for its anti-inflammatory and attenuated menopausal symptoms [9]. And it has been suggested that black cohosh may have chemo-preventive and anti-cancer potential [10]. The rhizomes and roots of the plant contain two major classes of secondary metabolites, triterpene glycosides and phenylpropanoids [11, 12]. The triterpene glycosides actein constitutes about 6.4% of an n-butanolic fraction of black cohosh enriched for triterpene glycosides (27%) [13]. Purified triterpene glycosides have been shown to inhibit the growth of various types of cancer cells *in vitro*, including human oral squamous carcinoma cells, breast cancer cells and liver cancer cells [14–16]. Triterpene glycosides from black cohosh have been shown to induce cell-cycle arrest at G1 [15]. Additionally, ACT could synergize with various kinds of chemotherapy agents at low doses to suppress the growth of cancer cells [17]. The growth-inhibitory effects of ACT may be associated with the altered expression of genes involved in the stress response pathways, the unfolded protein response and cell cycle control genes [18]. The bioactivity results for ACT should be helpful to elucidate its medical value to prevent human bladder cancer. Actually, it was the first time that ACT was investigated to indicate if it could inhibit the progression of bladder cancer and to reveal the underlying molecular mechanisms.

The cell cycle is associated with strict events, which modulate the cell division and proliferation through alterations of cyclin-dependent kinases (CDKs) and cyclins, leading to the transition in the process of cell cycle [19, 20]. During this event, dysfunctional expression of genes arrested or delayed checkpoints prior to cell division [21–23]. Thus, abnormal expression of CDKs or cyclins disorganized the process of cell division, contributing to the occurrence and progression of various tumors [24, 25]. And a variety of proteins, including p53, p21, and Cdc25C, play essential roles in controlling the expression of CDKs and Cyclins to regulate cell proliferation [26–28].

Autophagy is a lysosomal-dependent degradation process induced under various conditions or stresses [29]. Autophagy is reported as an alternative molecular mechanism by which the cell death is induced [30]. There is evidence that autophagy is needed for the death of cancer cells with defects in apoptosis [31]. In addition, new insights of the molecular mechanisms of autophagy are major reasons for the exploration of new potential drug targets [32, 33]. Induction of apoptosis presents an essential mechanism for drug-exploration against various cancers [34]. In general, there are two main apoptotic pathways: the intrinsic and the extrinsic [35]. The two pathways converge on the activation of Caspases, which contain a family of cysteine proteases and play an essential role in the completion of apoptosis [36, 37]. Accordingly, previous studies have pointed out that autophagy and apoptosis are tightly connected and may be modulated by ROS and JNK [38]. There is intriguing evidence that excessive ROS generation overcomes the cellular anti-oxidant defenses, inducing apoptosis [39]. Further, most cancer cells are more sensitive to rapid generation of ROS levels than normal cells [38, 40]. The phosphorylation of JNK is linked to ROS elevation [38, 41]. The JNK phosphorylation activated via ROS-dependent pathway triggers the over-expression of tumor suppressors, resulting in cell apoptosis [42]. Recently, AKT pathway is reported to suppress the formation of autophagy, and a large number of drugs are reported to treat the development of cancers via suppressing AKT phosphorylation.

In our study, the effects of ACT on human bladder cancer *in vitro* and *in vivo* were investigated. And the possible molecular mechanism by which the bladder cancer was suppressed was also explored, which were dependent on ROS/JNK- and AKT-regulated apoptosis and autophagy induction.

## RESULTS

### Actein suppresses cell proliferation in human bladder carcinoma cell lines

**Figure 1:**
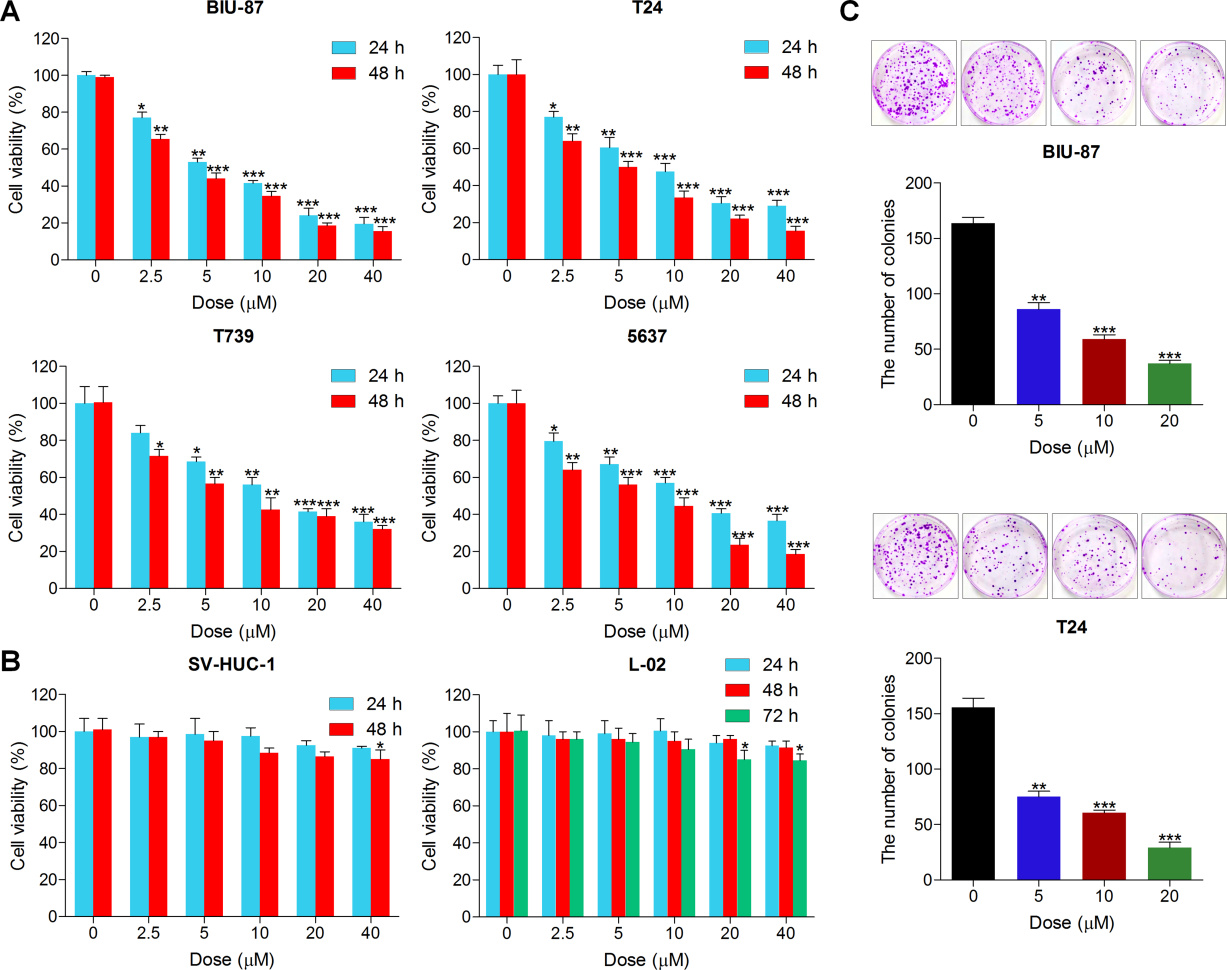
Actein suppresses cell proliferation in human bladder carcinoma cell lines (**A**) Human bladder cancer cell lines of BIU-87, T24, T739 and 5637 were treated with different concentrations (0, 2.5, 5, 10, 20 and 40 uM) of ACT for 24 h or 48 h, followed by MTT analysis to calculate the cell viability. (**B**) Human normal bladder cell line of SV-HUC-1 and human normal liver cell line of L-02 were cultured with ACT at the indicated doses for 24, 48 or 72 h, and then the cell viability was measured using MTT analysis. (**C**) Human bladder cancer lines of BIU-87 and T24 were treated with different doses of ACT for 24 h, followed by clonogenic assays. Data are represented as mean ± S.E.M. ^*^p < 0.05, ^**^p < 0.01, ^***^p < 0.001 versus the untreated group.

In order to explore the anti-proliferative effects of ACT on human bladder cancer, human bladder cancer cell lines, BIU-87, T24, T739 and 5637 were cultured with various concentrations of ACT for 24 and 48 h, followed by the assessment of cell viability using MTT analysis. As shown in Figure 1A, we found that the cell viability of human bladder cancer cells was dramatically down-regulated by ACT treatment in a dose- and time-dependent manner. Additionally, human normal bladder cell line of SV-HUC-1 and human normal liver cell line of L-02 were involved to further investigate the effects of ACT on non-cancer cell lines. From Figure 1B, SV-HUC-1 cells were not sensitive to ACT treatment, only at the treatment of highest dose of 40 uM for 48 h, significant difference was observed. Furthermore, administration of ACT for 72 h, both at 20 and 40 uM, exhibited relatively apparent difference compared to the control group without any treatment. Next, the cologenic assays were performed to calculate the role of ACT in regulating colony formation. The results indicated that ACT treatment considerably reduced the number of colonies of human bladder cancer cells in a dose-dependent manner (Figure 1C). The results above indicated that ACT suppressed the proliferation of human bladder cancer cells in a concentration- and time-dependent manner, exhibiting unconspicuous cytotoxicity to non-cancer cell lines, and that ACT might be used as a promising candidate against human bladder cancer.

### Actein induces G2/M cell cycle arrest in human bladder cancer cells

**Figure 2:**
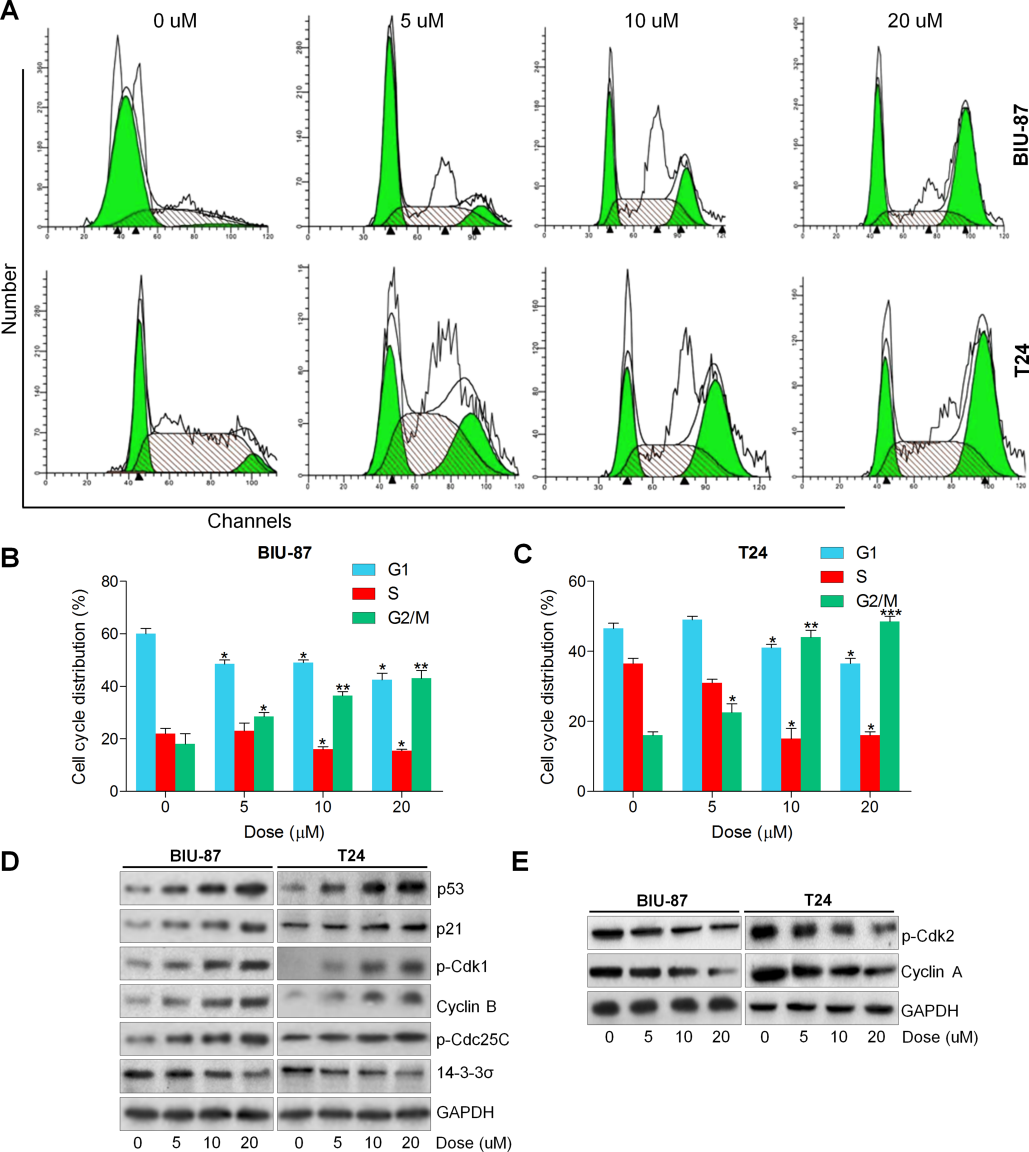
Actein induces G2/M cell cycle arrest in human bladder cancer cells (**A**) BIU-87 and T24 cells were cultured with ACT at the described concentrations for 24 h, and then flow cytometry analysis was used to calculate the cell cycle distributions. (**B**, **C**) The quantification of cell cycle arrest in ACT-treated BIU-87 and T24 cells were shown. (**D**) Signals associated with G2/M transition were investigated using western blot analysis. (**E**) Proteins involved in G1/S transition were measured through immunoblotting analysis. Data are represented as mean ± S.E.M. ^*^p < 0.05, ^**^p < 0.01, ^***^p < 0.001 versus the untreated group.

In this regard, to verify if the growth suppression caused by ACT is associated with cell cycle arrest, the role of ACT in the cell cycle distribution was measured. As shown in Figure 2A–2C, the proportion of bladder cancer cells at G1/S was significantly decreased after ACT treatment, while the percentage of cancer cells at G2/M phase was markedly increased owing to ACT treatment (0, 5, 10, and 20 uM) for 24 h. Subsequently, the cell cycle-associated molecules were evaluated using western blot analysis. The results exhibited that ACT enhanced p53, p21, p-Cdk1, Cyclin B and p-Cdc25C, while reduced 14-3-3σ in a dose-dependent manner, which were related to the regulation of G2/M cell cycle arrest (Figure 2D). In contrast, p-Cdk2 and Cyclin A were dose-dependently down-regulated by ACT, associated with the reduction of G1/S phase (Figure 2E). In conclusion, the findings above suggested that ACT induced G2/M cell cycle arrest through modulating the important signals of G2/M cell cycle transition-phase.

### Actein triggers autophagy in human bladder cancer cells

**Figure 3:**
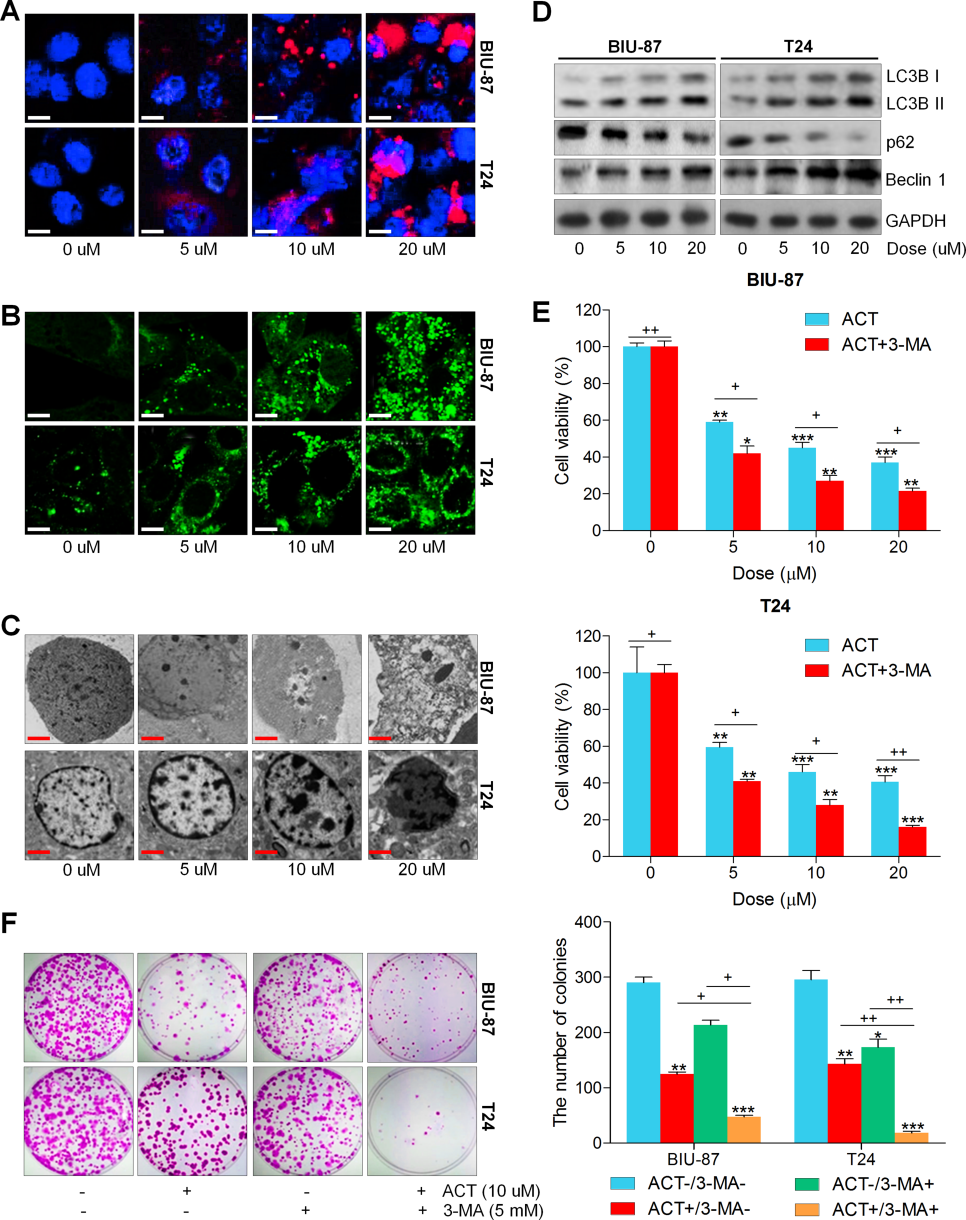
Actein triggers autophagy in human bladder cancer cells (**A**) Human bladder cancer cell lines of BIU-87 and T24 were exposed to ACT for 24 h. Then, all cells were harvested for LysoTracker Red DND-99 (50 nM) analysis to evaluate the cellular acidic compartments, revealing the autolysosomes and lysosomes situations. (**B**) BIU-87 and T24 cells were pre-transfected with GFP-LC3 plasmid for 24 h, followed by ACT administration for another 24 h at the described concentrations. And confocal microscope was used to observe the images of GFP-LC3 fusion proteins. The fluorescent intensity refers to the formation of autophagosomes. (**C**) BIU-87 and T24 cells were incubated with ACT for 24 h, and then all cells were collected for transmission electron microscopy (TEM) analysis. (**D**) Autophagy-related proteins, including LC3BI/II, p62 and Beclin 1, were assessed using western blot analysis. (**E**, **F**) BIU-87 and T24 cells were pre-treated with 3-MA (5 mM) for 2 h, followed by various ACT exposure for another 24 h. Then, all cells were collected for MTT and colony formation assays through cologenic analysis. Data are represented as mean ± S.E.M. ^*^p < 0.05, ^**^p < 0.01, ^***^p < 0.001 versus the untreated group; ^+^*p* < 0.05 and ^++^*p* < 0.01.

Autophagy is characterized by the enhanced acidic vesicular organelles, which has a close relationship with the formation of autophagosomes, and then the autophagosome fuses with the lysosome, forming autophagolysosome [23, 25, 28]. Enhancement of autophagic cell death from cancer cells is one of the best strategies in chemotherapy [27, 28]. Thus, we explored if or not ACT could trigger autophagy in human bladder cancer cells. At the beginning, the LysoTracker Red staining was used to mark the cellular acidic components, including autophagosomes and lysosomes. As shown in Figure 3A, the bladder cancer cells treated with ACT exerted more acidic vesicular organelles around the nucleus of cytoplasm in a dose-dependent manner. Following, the GFP-LC3 puncta transfection was applied to evaluate the condition of fluorescent puncta of autophagosomes. Figure 3B indicated that intensive GFP-LC3 puncta formation was observed in ACT-treated groups of bladder cancer cell lines. Next, the electric microscopy analysis revealed that ACT treatment significantly damaged bladder cancer cells that exhibited the shrunken state with an intact membrane, aggregated chromatin and pseudopodia-like protrusions, while the normal bladder cancer cells showed well-distributed chromatin and a clear nuclear membrane (Figure 3C). When cells undergo autophagic cell death, Microtubule-associated protein 1A/1B-light chain 3 (LC3)-II, an autophagosomal marker, increases from the conversion of LC3-I [43]. Western blot analysis indicated that LC3BI and LC3BII, and Beclin 1, involved in autophagy formation, were found to be highly induced by ACT culture in a dose-dependent manner, while p62 was greatly reduced (Figure 3D). According to previous studies, autophagy could protect the cell survival and induce cell death through various pathways [44]. In order to calculate if ACT-induced autophagy is a pro-death way or a pro-survival way, 3-MA, as essential autophagy inhibitor, was applied to pre-incubate the bladder cancer cells, followed by ACT treatment. Figure 3E suggested that administration with 3-MA enhanced the suppressive effect of ACT on bladder cancer cell viability. Similarly, the colony formation of human bladder cancer cells was dramatically inhibited by co-treatment with 3-MA (Figure 3F). Taken together, the data above indicated that ACT triggered autophagy in bladder cancer cells, which might be in a pro-survival way.

### Actein induces apoptosis in human bladder carcinoma cells

**Figure 4:**
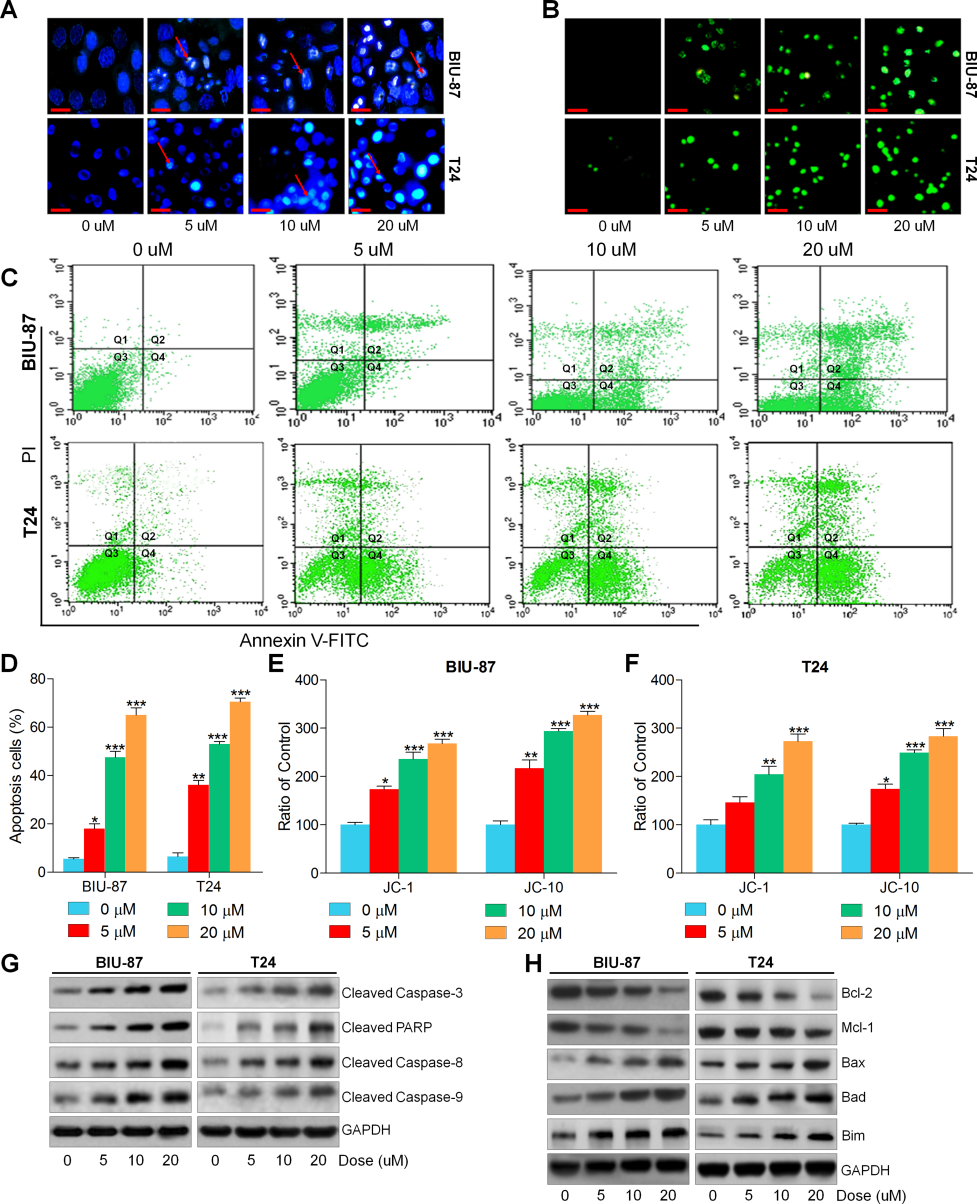
Actein induces apoptosis in human bladder carcinoma cells (**A**) BIU-87 and T24 cells were incubated with ACT for 24 h, followed by Hoechst 33258 analysis. (**B**) TUNEL assays were applied to explore the apoptosis in cells. (**C**) Bladder cancer cells were cultured with ACT for 24 h, and then analyzed through flow cytometry. (**D**) The apoptosis proportion of bladder cancer cells was shown following flow cytometry. (**E**, **F**) BIU-87 and T24 cells were treated with ACT for 24 h, and then the mitochondrial membrane potential was verified through measuring JC-1 and JC-10 levels. And the ratio of control was displayed. (**G**) Western blot analysis of cleaved Caspase-3, PARP, Caspase-8 and Caspase-9. (**H**) The western blot analysis of Bcl-2, Mcl-1, Bax, Bad and Bim. Data are represented as mean ± S.E.M. ^*^p < 0.05, ^**^p < 0.01, ^***^p < 0.001 versus the untreated group.

Apoptosis is a mode of cell death in which single cells are eliminated in the midst of living tissue, which is a key molecular mechanism by which anti-cancer drugs were investigated [36, 37, 40]. Hence, if apoptosis involved in the suppression of bladder cancer cells by ACT was further explored. As shown in Figure 4A and 4B, Hoechst 33258 staining and TUNEL analysis indicated that ACT significantly induced apoptosis along with nuclei fragmentation, chromatin condensation and cell shrinkage. And TUNEL-positive intensity through confocal microscope was highly enhanced by ACT administration. Moreover, flow cytometry analysis indicated that both early and late apoptosis cells were dose-dependently elevated by ACT treatment (Figure 4C and 4D). Furthermore, JC-1 and JC-10 levels were assessed to evaluate the mitochondrial membrane potential. Figure 4E and 4F revealed that the fluorescent intensities for both J-aggregates and monomeric forms of JC-1 and JC-10 were dramatically enhanced by ACT in a dose-dependent manner in both BIU-87 and T24 cells. Also, the western blot results indicated that cleaved Caspase-3, PARP, Casapse-8 and Caspase-9 were all considerably enhanced by ACT (Figure 4G). Also, anti-apoptotic and pro-apoptotic molecules were measured by immunoblotting analysis. Figure 4H indicated that B cell CLL/lymphoma 2 (Bcl-2) and Myeloid Cell Leukemia-1 (Mcl-1), as important anti-apoptotic signals, were reduced by ACT administration, while Bax, Bad and Bim were augmented dose-dependently, indicating pro-apoptosis. The results above revealed that cell apoptosis was induced by ACT through both intrinsic and extrinsic pathways.

### Actein-induced apoptosis is enhanced by suppressing autophagy

**Figure 5:**
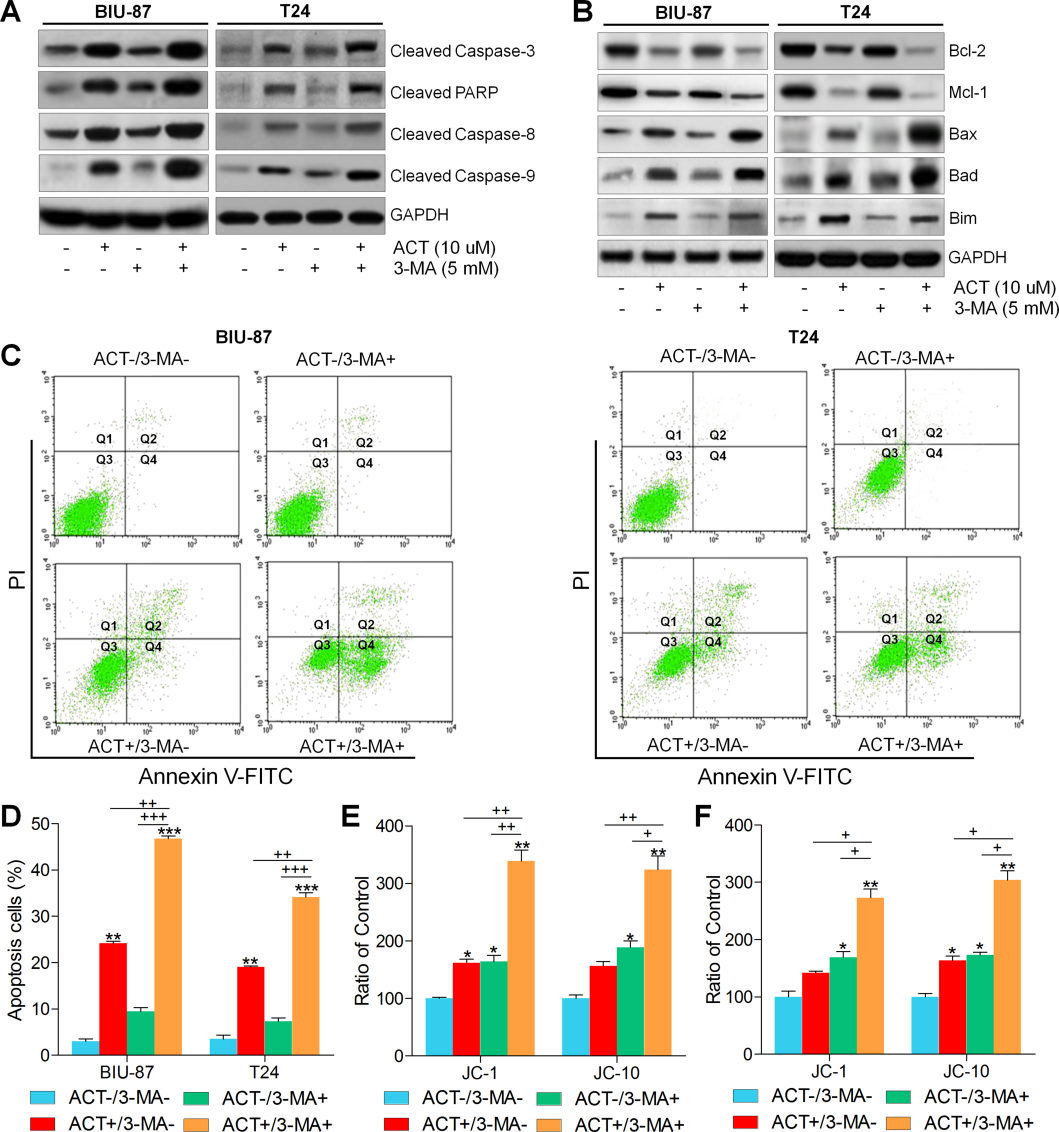
Actein-induced apoptosis is enhanced by suppressing autophagy BIU-87 and T24 cells were pre-incubated with 3-MA for 2 h, followed by ACT treatment for another 24 h. (**A**, **B**) Then, western blot analysis was used to evaluate the apoptosis-related signals. (**C**) BIU-87 and T24 cells were pre-treated with 3-MA for 2 h, followed by ACT exposure for another 24 h. Then, all cells were harvested for flow cytometry analysis to assess the apoptosis. (**D**) The quantification of apoptosis proportion was exhibited following the flow cytometry assays. (**E**, **F**) BIU-87 and T24 cells were treated as described, and then the mitochondrial membrane potential was measured through assessing JC-1 and JC-10 levels. The ratio of control was exhibited. Data are represented as mean ± S.E.M. ^*^p < 0.05, ^**^p < 0.01, ^***^p < 0.001 versus the untreated group; ^+^*p* < 0.05, ^++^*p* < 0.01 and ^+++^*p* < 0.001.

Previous studies indicated that autophagy could modulate apoptosis to infect the cell survival or death [45]. Therefore, here we further used 3-methyladenine (3-MA) combined with or without ACT to investigate the molecular mechanism. As shown in Figure 5A, we found that pre-treatment with 3-MA together with ACT significantly enhanced cleaved Caspase-3, PARP, Casapse-8 and Caspase-9. Reduced levels of Bcl-2 and Mcl-1 were observed in 3-MA/ACT groups. In contrast, Bax, Bad and Bim were highly induced by 3-MA and ACT co-culture (Figure 5B). Also, flow cytometry analysis directly demonstrated that both early and late apoptosis in bladder cancer cells were dramatically induced due to ACT and 3-MA co-treatment (Figure 5C and 5D). Finally, JC-1 and JC-10, indicating mitochondrial potential, were similarly found to be triggered owing to 3-MA/ACT treatment (Figure 5E and 5F). Thus, the results above indicated that treatment with 3-MA promoted ACT apoptotic effects. And the autophagy induced by ACT might be pro-survival.

### Actein potentiates ROS generation and JNK phosphorylation, and suppresses AKT pathway

**Figure 6:**
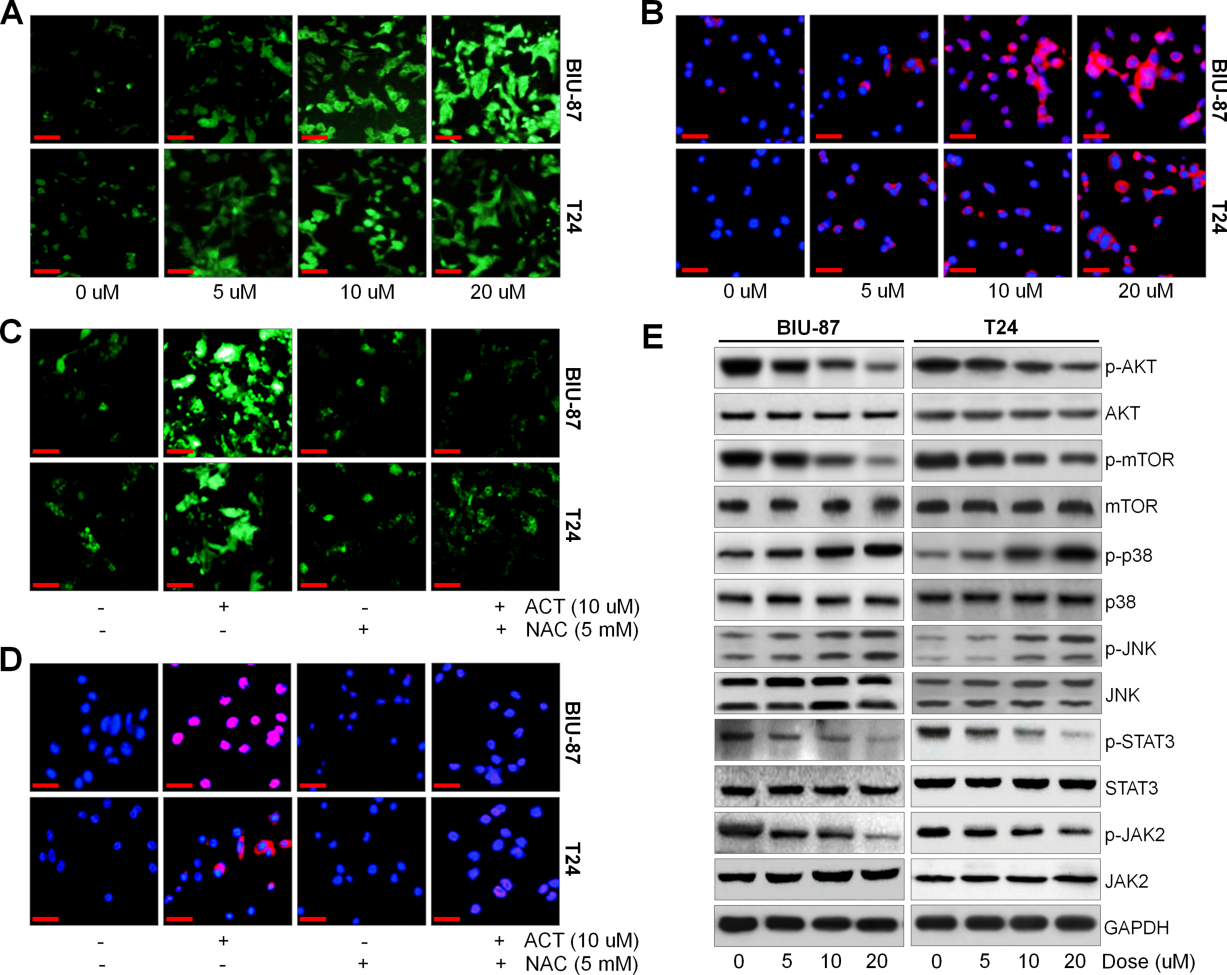
Actein potentiates ROS/JNK, and suppresses AKT pathway (**A**) BIU-87 and T24 cells were treated with different doses of ACT for 24 h, and then all cells were collected for DCF analysis to calculate the generation of ROS using fluorescent microscope. (**B**) BIU-87 and T24 cells were stained with MitoSOX Red after various treatments, and then the ROS levels were examined through confocal microscope. (**C**, **D**) The bladder cancer cells were pre-treated with ROS scavenger of NAC (5 mM) for 2 h, and then all cells were subjected with or without ACT (10 uM) for another 24 h, followed by DCF and MitoSOX Red staining using confocal microscope. (**E**) BIU-87 and T24 cells were treated with ACT for 24 h, and then p-AKT, p-mTOR, p-p38, p-JNK, p-STAT3 and p-JAK2 protein levels were evaluated using western blot analysis. Data are represented as mean ± S.E.M.

ROS generation is a crucial modulator in a variety of signaling pathways, which are associated with autophagy and apoptosis [38, 39]. Augment of DCF fluorescence in bladder cancer cells after ACT treatment was observed compared to the control group in the absence of ACT (Figure 6A). MitoSOX Red, an indicator of oxidation-sensitive red fluorescence, was used to further explore the role of ACT in regulating ROS generation. As shown in Figure 6B, we found that BIU-87 and T24 cells after ACT treatment exhibited dose-increased MitoSOX Red fluorescence, further indicating the elevation of ROS. Following, ROS scavenger of N-acetyl cysteine (NAC) was further treated to bladder cancer cells. Figure 6C indicated that ACT-induced ROS production was significantly reduced by NAC. Consistently, ACT-caused increased MitoSOX Red fluorescence was also scavenged by NAC (Figure 6D).

In order to further reveal the underlying molecular mechanism of the anti-cancer effects of ACT, the AKT and JNK signaling pathways were investigated using western blot analysis. AKT signaling pathway is considered to negatively modulate autophagy and apoptosis [46]. Figure 6E indicated that ACT dose-dependently reduced the AKT/mTOR and JAK2/STAT3 activity, while p38 and JNK were highly phosphorylated.

### The effects of ROS and p-JNK in cell cycle arrest, apoptosis and autophagy triggered by actein in human bladder cancer cells

ROS production is reported to be an essential regulator or inducer for apoptosis, autophagy and JNK activation to influence cell proliferation [47, 48]. As shown in Supplementary Figure 1A and 1B, NAC and JNK inhibitor of SP600125 were pre-treated to BIU-87 and T24 cells for 2 h, followed by ACT culture for another 24 h. And MTT analysis indicated that ACT-reduced cell viability was reversed for NAC pre-treatment, which was comparable to the ACT-treated groups. Additionally, SP600125 also reversed the cancer cell death caused by ACT in bladder cancer cells (Supplementary Figure 1B). Furthermore, flow cytometry assays suggested that both NAC and SP600125 significantly impeded ACT-induced G2/M cell cycle arrest, while induced G1/S phase arrest (Supplementary Figure 1C). And NAC showed more suppressive role in the induction of G2/M phase arrest for ACT treatment. Western blot analysis further revealed that both in BIU-87 and T24 cells, pre-incubation with NAC and SP600125 reversed the phosphorylated p38 and JNK levels induced by ACT, which was along with the reduced cleavage of Caspase-3, PARP, Casapse-8 and Caspase-9, as well as LC3BI/II, Beclin 1 and p53 (Supplementary Figure 1D and 1E). In conclusion, the data above indicated that ACT could induce ROS generation and activate JNK expression, whereas suppress AKT pathway, which might be linked to cell proliferation, apoptosis and autophagy triggered by ACT in human bladder cancer cells.

### AKT pathway is involved in cell cycle arrest, apoptosis and autophagy triggered by actein in human bladder cancer cells

On the contrary to the role of NAC as well as SP600125, AKT inhibitor, MK2206, significantly elevated the role of ACT in inducing bladder cancer cell death (Supplementary Figure 2A). Also, pre-treatment with rapamycin (RAPA), mTOR suppressor, ACT exhibited enhanced effects on the induction of cell death (Supplementary Figure 2B). Following, the flow cytometry analysis suggested that both MK2206 and RAPA dramatically up-regulated G2/M arrest, and down-regulated G1/S phase arrest in human bladder cancer cells ACT with ACT induction (Supplementary Figure 2C). In Supplementary Figure 2D and 2E, MK2206 and RAPA greatly enhanced apoptosis- and autophagy-associated proteins in bladder cancer cells either with or without ACT treatment. At the same time, MK2206 and RAPA observably promoted p38 and JNK phosphorylation induced by ACT. The findings above indicated that apoptosis and autophagy triggered by ACT in human bladder cancer cells could be potentiated by suppressing AKT signaling pathway.

### Actein inhibits the growth of human bladder xenograft mice *in vivo*

**Figure 7:**
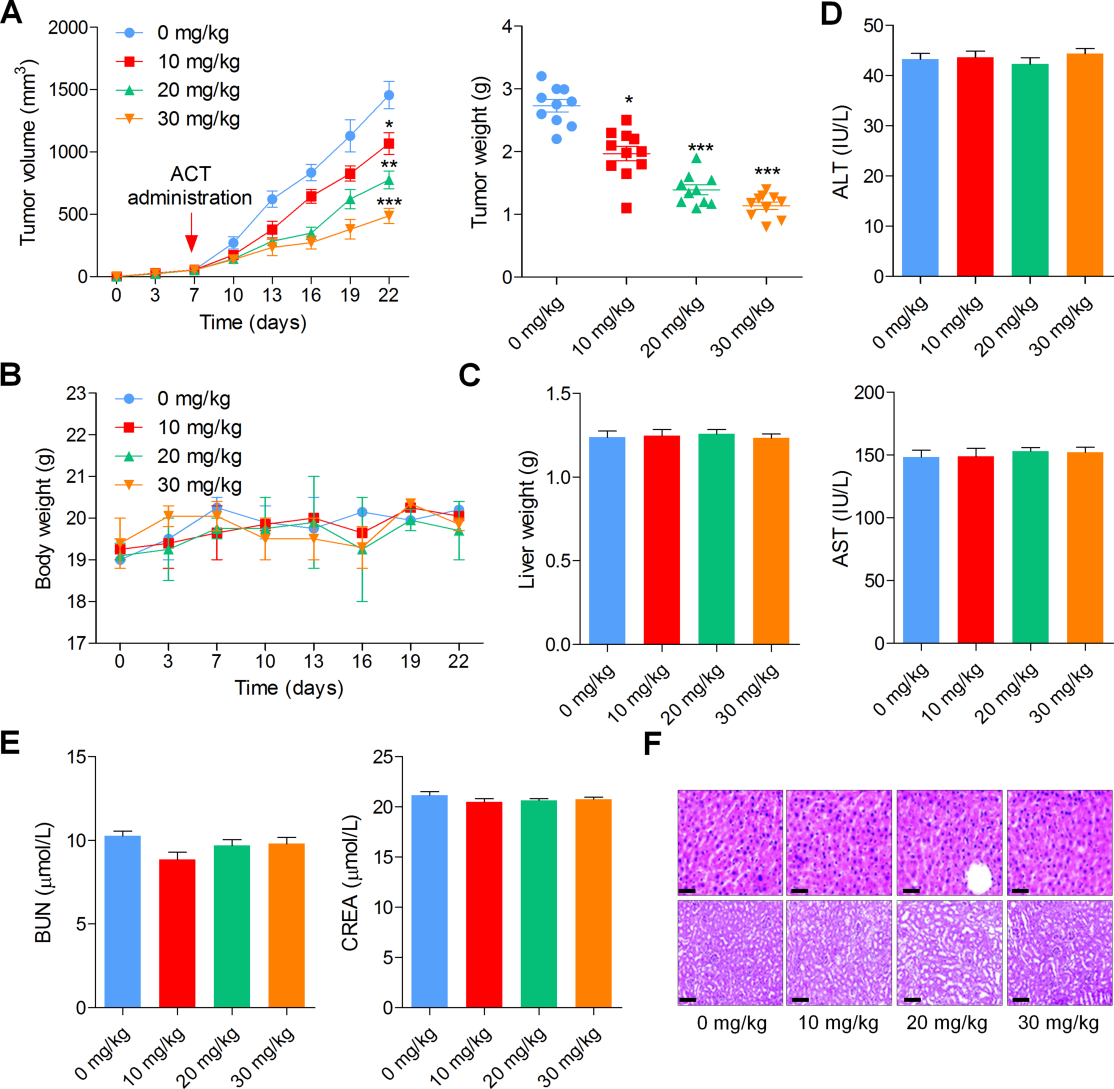
Actein inhibits the growth of human bladder xenograft mice *in vivo* 2 × 10^5^ BIU-87 cells were subcutaneously inoculated into nude mice. When tumors were obvious (tumor size about 50 mm^3^), mice were randomly grouped to receive 10, 20 and 30 mg/kg ACT for 15 days. (**A**) Tumor volume and tumor weight were measured. (**B**, **C**) The body weight and liver weight were examined. (**D**) The ALT and AST levels in plasma were determined to evaluate the hepatic toxicity. (**E**) The renal toxicity was calculated through measuring serum BUN and CREA levels in mice. (**F**) H&E staining of liver and kidney isolated from mice. Data are represented as mean ± S.E.M. ^*^p < 0.05, ^**^p < 0.01, ^***^p < 0.001 versus the untreated group.

In order to verify the anti-cancer role of ACT in bladder cancer cells *in vivo*, an intracranial nude mouse model was established using BIU-87 cells. BIU-87 cells were injected into the flanks of athymic nude mice to establish the xenograft tumors. When the tumor size reached to about 50 mm^3^, all mice were separated into four groups (0, 10, 20, and 30 mg/kg). As shown in Figure 7A, the growth of tumor was markedly reduced by ACT administration in a dose-dependent manner, evidenced by tumor size and tumor weight measurement. Meanwhile, there was no significant alteration in body weight and liver weight among different groups of mice (Figure 7B and 7C). In addition, serum ALT and AST, indicating hepatic toxicity, in mice treated with different doses of ACT were found to be similar in each group (Figure 7D). Figure 7E indicated that there was no obvious difference of BUN and CREB in ACT-treated groups. Finally, H&E staining suggested that ACT administration exhibited no histology changes in each group of mice (Figure 7F). The results here indicated that ACT suppressed human bladder cancer progression *in vivo* with low toxicity to animal bodies.

**Figure 8:**
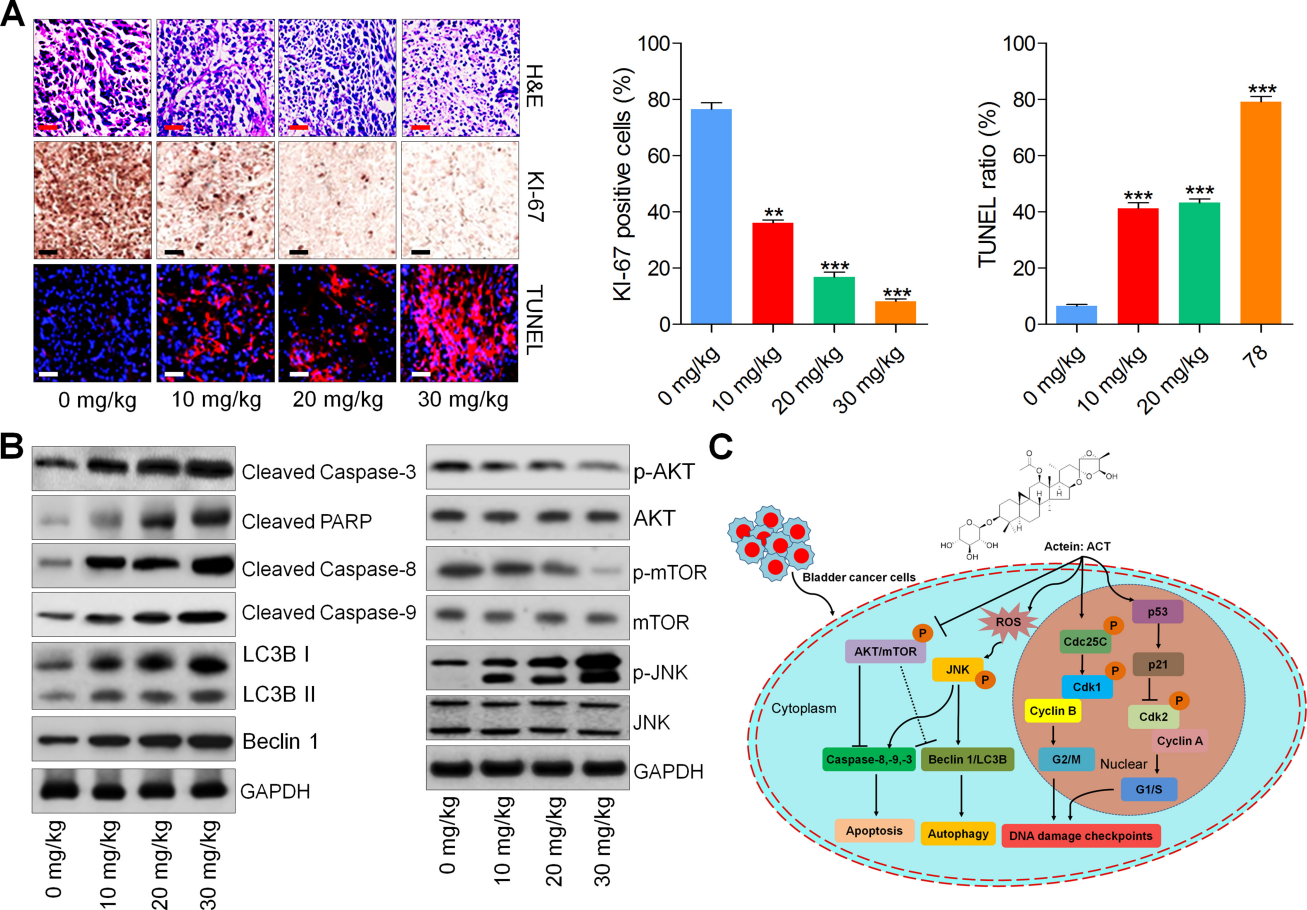
Actein-suppressed growth of human bladder xenograft mice is associated with induction of apoptosis and autophagy (**A**) H&E staining, KI-67 and TUNEL expression levels of tumor tissues were measured using IHC analysis. (**B**) Western blot analysis of Cleaved Caspase-3, PARP, Caspase-8, Caspase-9, LC3BI/II, Beclin 1, p-AKT, p-mTOR, and p-JNK in tumor tissue samples. (**C**) The proposed work model of ACT in suppressing human bladder cancer cells. ACT could inhibit cell proliferation through causing G2/M cell cycle arrest, and inducing apoptosis and autophagy by potentiating ROS and JNK activity, while inhibiting AKT pathway. Data are represented as mean ± S.E.M. ^*^p < 0.05, ^**^p < 0.01, ^***^p < 0.001 versus the untreated group.

H&E staining of tumor tissue sections indicated that ACT reduced the number of tumor cells. And the immunohistochemical analysis suggested lower levels of KI-67 positive cells while higher levels of TUNEL-positive cells in ACT-treated tumors, which were comparable to the control ones (Figure 8A and 8B). In addition, western blot analysis demonstrated that ACT improved the expression levels of cleaved Caspase-3, PARP, Casapse-8 and Caspase-9, as well as LC3BI/II and Beclin 1, which are associated with apoptosis and autophagy. p-AKT and p-mTOR were dose-dependently reduced by ACT, whereas JNK phosphorylation was elevated in tumor tissue segments (Figure 8B). In conclusion, the data above indicated that ACT suppressed human bladder cancer growth *in vivo* through inducing apoptosis and autophagy.

## DISCUSSION

Bladder cancer is a highly prevalent tumor and is related to substantial morbidity, mortality and cost [1, 2, 49]. The environmental or occupational exposures to carcinogens, particularly tobacco, are the major risk factors for promoting the bladder cancer [50, 51]. Currently, despite huge advances have been made, the therapeutic strategies for human bladder cancer are still limited. Thus, it is necessary to find new and novel treatments to prevent human bladder cancer progression. Actein is a bioactive triterpene glycoside, which is isolated from Cimicifuga species, and has been revealed for its suppressive effects on the growth and proliferation of breast cancer cells and osteoblastic cells [9, 10, 19, 20]. Considering its effective role in preventing cancer cells, as well as its long historic application in Asia, actein was used in our study to further reveal its anti-cancer ability. As we know, it was the first time that ACT was investigated in human bladder cancer. Here, we further found that ACT could inhibit cell proliferation, induce G2/M phase and reduce G1/S phase, and trigger autophagy and apoptosis in human bladder cancer cells. Of note, ACT at the same time potentiated ROS generation and JNK activation, while impeded AKT and mTOR activity both *in vitro* and *in vivo*, which was tightly involved in the induction of apoptosis and autophagy. In *vivo* experiments further confirmed that ACT greatly suppressed the tumor growth of nude mice bearing BIU-87 cells.

Dysfunction of cell cycle arrest is an essential marker for cancer progression [19–21, 52]. Many anti-cancer agents reduce malignant growth by arresting the cell cycle at the G1/S or G2/M phases [53]. According to previous studies, cell cycle arrest, particularly G2/M phase, might be a useful therapy to prevent the proliferation of cancer cells [54]. The CDK1, Cyclin B and Cdc25C are essential for inducing G2/M cycle phase, which could enhance the dividing of a cell into two [55]. Our study indicated that ACT administration improved p-Cdk1, Cyclin B, p-Cdc25c, p53 and p21 expression, while reduced 14-3-3σ levels, which are vital signals involved in G2/M phase arrest induction. P21 plays an important role in suppressing Cdk1, Cyclin B, Cdk2 and Cyclin A activation, which might be relied on p53 expression [56]. The p-Cdc25C was occurred, which was an up-streaming signal of Cdk1/Cyclin B, resulting in G2/M phase arrest. And Cdk2/Cyclin A induced G1/S phase cycle [57]. Both G2/M and G1/S phase cycle arrest are hallmarks of DNA damage checkpoints [58]. Decreased p-Cdk2 and Cyclin A were observed in ACT-treated bladder cancer cells. Thus, ACT triggered G2/M arrest and disturbance of G1/S were possibly associated with the improvement of p-Cdk1, Cyclin B, p-Cdc25c, p53 and p21, as well as the reduction of p-Cdk2 and Cyclin A.

Autophagy plays an important role in determining the cell condition [23]. Autophagic flux is the complete mechanism of autophagy originating with the fusion of the autophagosome with a lysosome, resulting in degradation and recycling of the cargo [30–32]. LC3 is necessary during proteolytic processing, yielding a 16-kDa LC3-I protein that conjugates with phosphatidyl ethanolamine to yield a 14-kDa LC3-II form, in which LC3-II is used as a marker of complete activation of autophagosome [43, 59, 60]. The p62 protein is involved in the lysosome-dependent degradation system that directly interacts with LC3-II and degradation in the process of autophagy, and suppression of autophagy results in enhanced p62 protein levels [44, 61]. Accumulating evidence suggested the dual effect of autophagy on cancer, contributing to cell death or protecting cell survival [31, 33–35]. In our study, we found that ACT induced autophagy dose-dependently, which was proved by the accumulation of autophagic vesicles and improvement of LC3B-I/II and Beclin 1, as well as reduction of p62. In addition, an autophagy inhibitor of 3-MA was used in our study, and 3-MA could accelerate the suppressive effects of ACT on the bladder cancer cell viability, indicating that ACT-triggered autophagy might be pro-survival.

Apoptosis is defined as programmed cell death PCD, executed by a family of cysteinyl aspartate proteinases known as Caspases [62]. Caspases are included in a family of highly conserved aspartate-specific cysteine proteases, which are expressed as inactive zymogens in animal cells as well as serve as the essential executioners of apoptosis [63, 64]. When death receptors are stimulated, the extrinsic pathway, involved in the activation of Caspase-8, is initiated. The intrinsic pathway includes the activation of caspase-9, and this process is regulated by B-cell lymphoma-2 (Bcl-2) family proteins, including the anti-apoptotic signals of Bcl-2 and Mcl-1, and the pro-apoptotic molecules of Bax, Bad and Bim [65, 66]. Once Caspases-3 is activated, it cleaves death substrates or a variety of proteins. During apoptosis, Caspase-3 cleaves PARP, decreasing its DNA repair activity and contributing to apoptotic cell death [62, 67]. Our results indicated that mitochondrial membrane potential was significantly decreased for ACT exposure. Mitochondrial membrane depolarization could result in the release of cytochrome c and activate Caspase-9. We also found that Caspase-3, PARP, Caspase-8 and Caspase-9 were highly cleaved due to ACT administration, accompanied with reduced Bcl-2 and Mcl-1 as well as elevated Bax, Bad and Bim expressions, suggesting that ACT-induced apoptosis was Caspases-dependent.

ROS generation has been reported to be important in regulating autophagy and apoptosis [41, 68]. Consistently, in our study ROS production was highly induced by ACT, which was involved in ACT-triggered autophagy and apoptosis. Additionally, ACT-caused high expression of cleavage of Caspase-3, PARP, Caspase-8, and Caspase-9 were significantly reversed by NAC, a ROS scavenger. Similarly, autophagy-related LC3BI/II and Beclin 1 expressions were abolished by NAC in ACT-treated bladder cancer cells. The findings here suggested that ACT-induced autophagy and apoptosis was dependent on ROS production. Recently, mitogen-activated protein kinases (MAPK s), and PI3K/AKT signaling pathways are closely involved in autophagy and apoptosis progression [69, 70]. JNK, and p38, main groups of MAPKs family, modulate a large number of cellular processes, including autophagy and apoptosis. P38 and JNK activity could positively induce apoptosis and autophagy [71]. In contrast, PI3K/AKT pathway was suggested to negatively modulate autophagy progression via controlling the mTOR phosphorylation [72, 73]. Further, JAK2/STAT3 signaling pathway is involved in the sustaining of self-renewal and tumorigenicity, which is constitutively phosphorylated in cancer cells [74, 75]. In our present study, ACT dramatically activated p38 and JNK phosphorylation, whereas inactivated AKT/mTOR and JAK2/STAT3 signaling pathways. Additionally, the relationship between JNK and AKT/mTOR with autophagy and apoptosis induced by ACT was further explored. The findings indicated that inhibiting the activity of JNK greatly eliminated cleaved Caspase-3, PARP, Caspase-8, and Caspase-9, as well as LC3BI/II and Beclin 1, which was induced by ACT. In contrast, inhibition of AKT and mTOR activation strengthened apoptosis- and autophagy-related signals expression. Thus, autophagy and apoptosis induced by ACT could be modulated by ROS/JNK, and AKT/mTOR pathways.

*In vivo*, ACT could inhibit the bladder tumor growth. And ALT, AST, BUN, and CREA showed no significant difference among various groups of mice, indicating that ACT at the concentrations used in our study, showed no hepatic and renal toxicity. And H&E staining of liver and kidney further confirmed the safety of ACT for application. Moreover, TUNEL assays indicated that apoptosis was induced by ACT *in vivo*. And western blot analysis illustrated that cleavage of Caspase-3, PARP, Caspase-8, and Caspase-9 were significantly induced by ACT, and LC3B-I/II and Beclin 1 were also potentiated due to ACT treatment in tumor samples.

Together, our findings exhibited that ACT inhibited the cell proliferation, induced autophagy and apoptosis that were dependent on ROS/JNK activity, and AKT inactivity in human bladder cancer cells. Inhibiting autophagy potentiated ACT-triggered cell death and apoptosis, revealing that autophagy induced by ACT was a pro-survival procedure. The working model of ACT in suppressing human bladder cancer development was exhibited in Figure 8C. In conclusion, our study indicated that ACT might be a promising candidate against human bladder cancer development.

## MATERIALS AND METHODS

### Materials

3-(4,5-dimethyl-2-thiazolyl)-2,5-diphenyl-2H-tetrazolium bromide (MTT) were purchased from the Sigma Chemical Company (St. Louis, MO). The primary antibody of GAPDH (dilution: 1:200) was purchased from Santa Cruz Biotechnology (USA). p53, p21, p-Cdk1, Cyclin B, p-Cdc25C, 14-3-3σ, p-Cdk2, Cyclin A, LC3B, p62, Beclin 1, PARP, Bcl-2, Mcl-1, Caspase-9, Caspase-8, Caspase-3, Bax, Bim, p-STAT3, STAT3, p-JAK2 and JAK2 were purchased from Abcam (dilution: 1:1000, USA). Bad, p-p38, p38, p-AKT, AKT, p-mTOR, mTOR, JNK and p-JNK were obtained from Cell Signaling Technology (dilution: 1:1000, USA). The secondary horseradish peroxidase (HRP)-conjugated antibodies were purchased from Jackson ImmunoResearch (West Grove, PA, USA). Actein was purchased from ChromaDex (Laguna Hills, CA), which was purified by high-performance liquid chromatography (HPLC). Annexin V-FITC/PI Detection kit was obtained from BioVision (CA, USA). Alanine aminotransferase (ALT) Assay Kit, Aspartate aminotransferase (AST) Assay Kit, Urea (BUN) Assay Kit and Creatinine (Cr) Assay kit were purchased from Nanjing Jiancheng Bioengineering Institute (Nanjing, China). Dimethyl sulfoxide (DMSO) was obtained from Sigma-Aldrich Chemical Company. The enhanced chemiluminescence (ECL) and bicinchoninic acid (BCA) were purchased from Thermo Fisher Scientific (San Diego, CA, USA). The In situ BrdU-Red DNA Fragmentation (TUNEL) Assay Kit was purchased from Abcam. Annexin V/PI Cell Apoptosis Detection Kit was obtained from KeyGen Biotech (Shanghai, China). Hoechst 33258 was purchased from Invitrogen (Carlsbad, CA). JC-10 Mitochondrial Membrane Potential Assay Kit (Microplate) and JC-1 Mitochondrial Membrane Potential Assay Kit were purchased from Abcam. Reactive Oxygen Species Assay Kit was obtained from KeyGen Biotech. ROS scavenger of NAC, JNK inhibitor of SP600125, autophagy suppressor of 3-MA, and AKT inhibitor of MK2206 and mTOR inhibitor of Rapamycin (RAPA) were purchased from Sigma Chemical Company. LysoTracker Red DND-99 was purchased from Invitrogen (USA). One Step TUNEL Apoptosis Assay Kit was obtained from Beyotime.

### Cells and culture

Human bladder cancer cell line, BIU-87, was purchased from American Type Culture Collection (ATCC, Manassas, VA, USA). Human bladder cancer cell lines of 5637 and T24, human urothelial cell line of SV-HUC-1 and human normal cell line of L-02 were purchased from the Cell Bank of the Chinese Academy of Sciences (Shanghai, China). BIU-87, 5637 and L-02 were maintained in RPMI-1640 medium (GibcoBRL, Grand Island, NY, USA) containing 10% (w/v) fetal bovine serum in a humidified CO_2_ incubator under 5% CO_2_. SV-HUC-1 and T24 were cultured in F-12 medium (GibcoBRL). The cells were harvested in a 0.025% trypsin-EDTA (GibcoBRL) with phosphate buffered saline (PBS) solution, plated at the required cell number, and allowed to adhere before drug treatment. The cells were exposed to different doses of drugs for study.

### Cell viability analysis

MTT was used to calculate cell viability. 2 × 10^4^ cells/well were seeded on 96-well plates and incubated at 37°C overnight under an atmosphere of 95% air and 5% CO_2_, and then treated with various concentration of ACT (0, 2.5, 5, 10, 20 and 40 uM) for 24 h. MTT solution (300 μL/well) was added after incubation. Following incubation for an additional 4 h at 37°C, the supernatants were removed and 200 μl DMSO was added into each well to dissolve the formazan crystals. The 96-well plates were then placed in a microplate reader (Bio-Tek, USA) to assess the absorbance at 490 nm. Each test performed in triplicate.

### Colony formation assays

1000 cells, BIU-87 and T24, were planted into a 35-mm petri dish to adhere overnight. Following, a different ACT treatment was performed to the dishes for 24 h, after which the medium was replaced with drug-free medium. Cells were further cultured for two weeks to allow colonies formation. Next, the colonies were fixed with paraformaldehyde (4%) and then stained with crystal violet solution (0.1%), and washed, and imaged finally. The number of colonies larger than 0.5 mm in diameter was counted using a microscope (Nikon, Japan) at 400 × magnification.

### Flow cytometry analysis

The cells were harvested after treatment with ACT as indicated and stained with the Annexin V/PI Cell Apoptosis Detection Kit following the manufacturer's instructions. The cells in early stages of apoptosis were Annexin V positive and PI negative, and the cells in the late stages of apoptosis were both Annexin V and PI positive. The results acquisition and analysis were carried out with a Becton Dickinson FACS Calibur flow cytometer using Cell-Quest software (Becton Dickinson).

The effect of ACT, NAC, SP600125, MK2206 and RAPA treatment on cell cycle distribution was also measured using flow cytometry analysis. After treatment under various conditions, all cells were harvested and fixed in ethanol (70%). The cells were then rinsed with PBS for twice and stained with PI solution containing PI (50 μg/mL) and RNAse (25 μg/mL) for 30 min. In the end, cells were analyzed on a FACS Calibur flow cytometer and analyzed with Cell-Quest Pro software.

### TUNEL assays *in vitro*

The bladder cancer cells were exposed to ACT administration for 24 h on 12-well plates. Then, One Step TUNEL Apoptosis Assay Kit was used to evaluate apoptosis following the manufacturer's instructions. Nuclei were stained with 4′,6-diamidino-2-phenylindole (DAPI). The staining intensity was measured by a fluorescence microscopy.

### Hoechst 33258 analysis

The bladder cancer cells after ACT administration for 24 h on 12-well plates were then stained with 10 μg/ml of Hoechst 33258 in 1ml PBS for 30 min. Hoechst 33258 was applied for nuclei staining of cells. After staining, samples were rinsed with PBS once and 1ml of PBS was added. The representative images of cells were captured using a fluorescence microscopy.

### Mitochondrial potential assessment

JC-1 and JC-10 dye loading solutions were added to BIU-87 and T24 cells after treatment as indicated and incubated for 30 minutes. The fluorescent intensities for both J-aggregates and monomeric forms of JC-1 and JC-10 were measured at Ex/Em = 490/525 nm and 490/590 nm with a microplate reader (Biotek, Winooski, VT).

### Western blot analysis

Human bladder cancer cells after different treatments were harvested and washed with chilled PBS and harvested in sample buffer (150 mM NaCl, 100 mM NaF, 50 mM Tris-HCl (pH 7.6), 0.5% Nonidet P-40 (NP-40) and 1 mM PMSF). And the bladder tumors were homogenized into 10% (wt/vol) hypotonic buffer (pH 8.0, 1mM EDTA, 5 μg/ml leupeptin, 25mM Tris-HCl, 1mM Pefabloc SC, 5 μg/ml soybean trypsin inhibitor, 50 μg/ml aprotinin, 4mM benzamidine) to yield a homogenate. Then, the final supernatants from cells and tumors were obtained by centrifugation at 14,000×g for 20 min at 4°C. Protein concentration was determined using BCA protein assay kit with bovine serum albumin as a standard. Sample-loading buffer was added, the mixture was boiled for 5 min. And the total protein extract are used for Western blot analysis. 40 μg of total protein was loaded and proteins were separated using 10% SDS-PAGE and electrophoretically transferred to the polyvinylidene difluoride membranes (Millipore, USA). The membranes were then blocked with 5% skim milk Tris buffered saline with 0.1% Tween 20 (TBST), washed, and then incubated with primary antibody overnight at 4°C. Then, the membrane was washed with TBST for three times, followed by incubation with a horseradish peroxidase (HRP)-conjugated secondary antibody (1:2500) at room temperature for 2h. Following another round of washing with TBST, the membrane was then developed using ECL, and exposed to Kodak (Eastman Kodak Company, USA) X-ray film. Every protein expression levels will be defined as grey value using ImageJ 1.38 software (National Institutes of Health, USA) and standardized to housekeeping gene of GAPDH and expressed as a fold of control. All experiments were performed in triplicate and done three times independently.

### Measurement of ROS generation

Intracellular ROS production was measured using the Reactive Oxygen Species Assay Kit. The bladder cancer cells were plated in six-well plates at a density of 5 × 10^5^/ml and exposed to ACT and NAC, or the two in combination as indicated in each part. Cells were then stained with DCFH-DA (10 μM) in the dark room for 30 min at 37 °C. Next, the cells were rinsed with serum-free DMEM three times, and the level of ROS generation was detected using fluorescence microscopy.

### LysoTracker red assays

5 × 10^5^/ml bladder cancer cells were seeded in six-well plates and treated with ACT and NAC, or the two in combination as indicated for 24 h. Cells were then incubated with LysoTracker Red DND-99 (50 nM) in the dark room at 37°C for 30 min. Finally, the immunofluorescence images were captured using a confocal microscope.

### Transmission electron microscopy (TEM) analysis

After different treatments with ACT as indicated for 24 h, the cells were collected and washed with PBS then fixed in 2.5% glutaraldehyde overnight. Then, the cells were washed by PBS (0.1 M) and fixed with OsO4 (1%). Following, the cancer cells were dehydrated with a range of alcohol concentrations for 15 minutes. The cells were then embedded into paraffin and sliced using an LKB-V ultramicrotome (LKB, Stockholm, Sweden). For TEM images acquisition, the prepared sections were observed on a JEM-2100 microscope operating at microscope at an accelerating voltage of 200 kV (JEOL Ltd., Tokyo, Japan).

### GFP-LC3 puncta analysis

In order to calculate the formation of fluorescence puncta of autophagosomes, BIU-87 and T24 cells were transfected with GFP-LC3 plasmid. Then, all cells were cultured with 0, 5, 10 and 20 μM ACT for 24 h. Next, the cells were rinsed with PBS twice, fixed with 4% paraformaldehyde for 20 min, and permeabilized with 0.1% Triton X-100. Finally, the representative images were obtained using a fluorescent microscope.

### Human bladder cancer xenograft experiment

4-week-old, female, BALB/c athymic nude mice (nu/nu) were purchased from Shanghai Slac Laboratory Animal Company Limited (Shanghai, China). Mouse care and usage were performed following the local ethical guidelines. The mice were raised in air-conditioned pathogen-free rooms under controlled lighting (12 h light/day) and fed with water and standard laboratory food. All protocols were in accordance with the Regulations of Experimental Animal Administration issued by the Ministry of Science and Technology of the People's Republic of China. The animal study was carried out in accordance with the regulations of The Fourth Military Medical University (Xian, Shanxi, China). After 1 week's acclimation, single-tumor cell suspensions (BIU-87, 2 × 10^5^) were subcutaneously injected into the left flank of each mouse to obtain bladder cancer xenografts. The mice were divided into four groups (15 mice/group) 7 days after cell implantation. Mice in the experimental groups were intraperitoneally injected with ACT at a dose of 0, 10, 20 and 30 mg/kg body weight per day. Mice in the control group received an equal volume of normal saline. The tumor volume was calculated every two days by two cross-sectional measurements, and the tumor size was measured as followings: tumor volume = width^2^ × length × 0.4. Mice were sacrificed after 22 days, and the tumors were weighed and then fixed in 10% formalin for the following experiments. The blood of each mouse was extracted through eyeballs and the serum was separated by centrifuge at 3,000×g for 15 min. Then, the activities of ALT, AST, BUN and CREA in serum were evaluated following the manufacturer's protocol. At the end of the experiment, all rest of mice were sacrificed. The liver, and renal tissues were excised, weighed, and fixed in 10% formalin for histology analysis.

### Immunohistochemical analysis

Formalin-fixed tissue samples were embedded in 4% paraffin and then the paraffin-embedded specimens were cut into serial sections (3-μm thickness). Primary tumors, liver, lung, and renal sections were stained with hematoxylin and erosion (H&E). Mouse bladder tumors were sectioned at 3 μm thickness, and stained with KI-67 (Abcam, 1:200). The tumor sections were analyzed using a microscope. Images were arranged through TissueFAXs (Tissue-Gnostics) software. The percentage of KI-67-positive cells in each tumor section was quantified using the HistoQuest software. The number of KI-67-positive cells was divided by the total number of cells in each tumor section. The apoptosis of tumor tissues was evaluated using TUNEL assay with the In situ Apoptosis Detection Kit following the manufacture's protocol. After deparaffinization and hydration, tumor tissue sections were washed with PBS twice and then incubated with proteinase K (20 μg/ml, Abcam) for 25 min at 37°C, followed by washes with PBS. Then, all sections were incubated with TUNEL mixture. The tumor sections were counter-stained with DAPI. Finally, the tissue sections were observed with a confocal microscopy.

### Statistical analysis

Results are expressed as the mean ± SEM of triplicate experiments. Statistically significant values were compared using the ANOVA and the Dunnett's *post-hoc* test, and *P*-values of <0.05 were considered to indicate a statistically significant result.

## SUPPLEMENTARY MATERIALS FIGURES


