# Supplementary material for: Actein induces autophagy and apoptosis in human bladder cancer by potentiating ROS/JNK and inhibiting AKT pathways
Source: Oncotarget. 2017 Nov 1;8(68):112498–515. doi: 10.18632/oncotarget.22274 (PMC5762528; doi:10.18632/oncotarget.22274)
Supplement: Supplementary file 1 [file oncotarget-08-112498-s001.pdf]

Actein induces autophagy and apoptosis in human bladder cancer by potentiating ROS/JNK and inhibiting AKT pathways

SUPPLEMENTARY MATERIALS

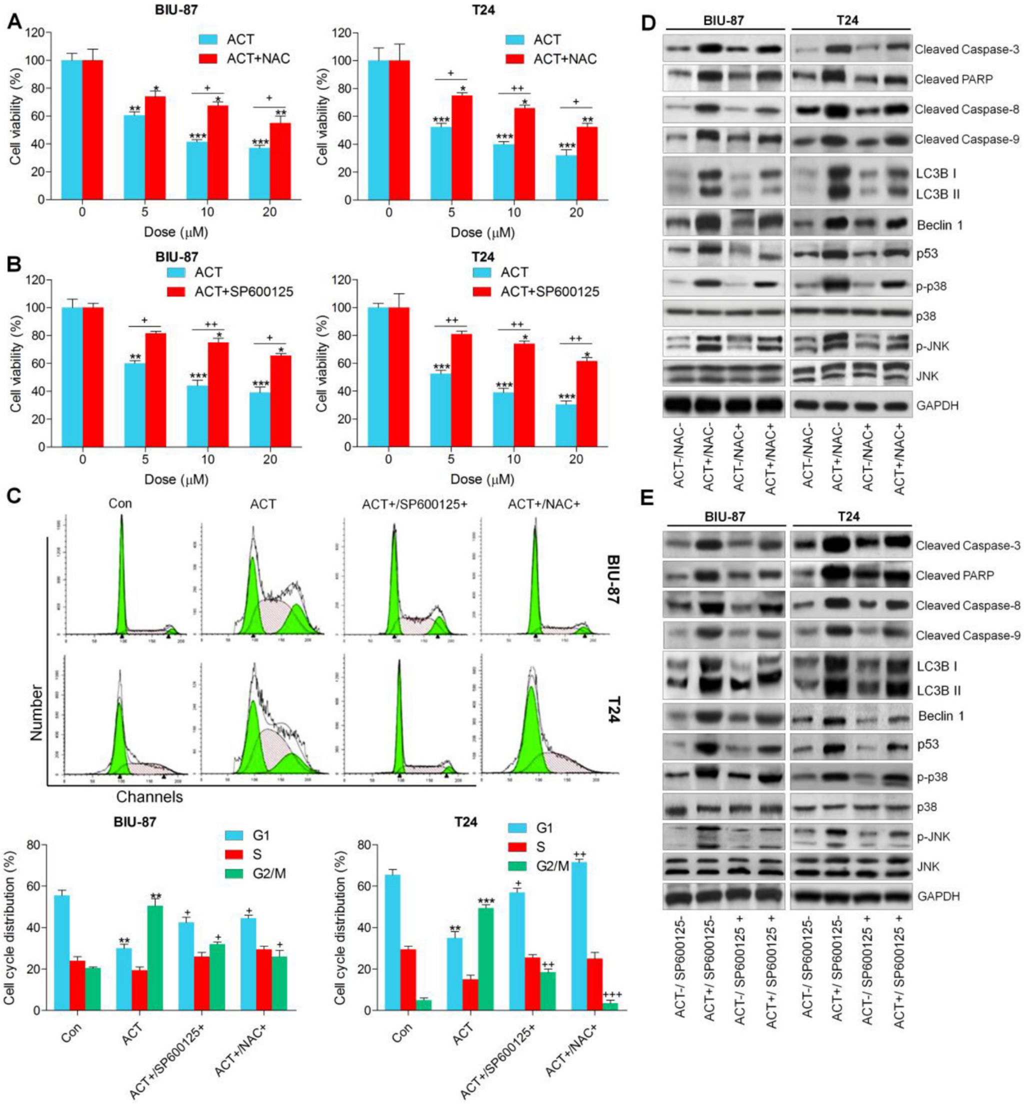

Supplementary Figure 1: The effects of ROS and p-JNK in cell cycle arrest, apoptosis and autophagy triggered by actein in human bladder cancer cells. (**A**) BIU-87 and T24 cells were pre-incubated with NAC for 2 h, which was followed by ACT administration at the indicated concentrations for 24 h. Subsequently, MTT analysis was used to calculate the cell viability. $^{+}p < 0.05$, $^{++}p < 0.01$ and $^{+++}p < 0.001$. (**B**) Bladder cancer cells were pre-treated with JNK inhibitor of SP600125 (5 mM) for 2 h, and then subjected to ACT for another 24 h. Next, the cell survival was measured using MTT assays. (**C**) BIU-87 and T24 cells were treated as described, and then all cells were harvested for flow cytometry to calculate the cell cycle arrest. The cell cycle distribution was quantified and exhibited in histogram. $^{+}p < 0.05$, $^{++}p < 0.01$ and $^{+++}p < 0.001$ versus the ACT group. (**D**) BIU-87 and T24 cells were pre-treated with NAC for 2 h, followed by ACT administration for further 24 h. And then, all cells were collected for western blotting analysis. (**E**) BIU-87 and T24 cells were pre-cultured with SP600125 for 2 h, and then subjected to ACT for another 24 h. Next, all cells were harvested for western blot. Data are represented as mean ± S.E.M. $^{*}p < 0.05$, $^{**}p < 0.01$, $^{***}p < 0.001$ versus the untreated group.

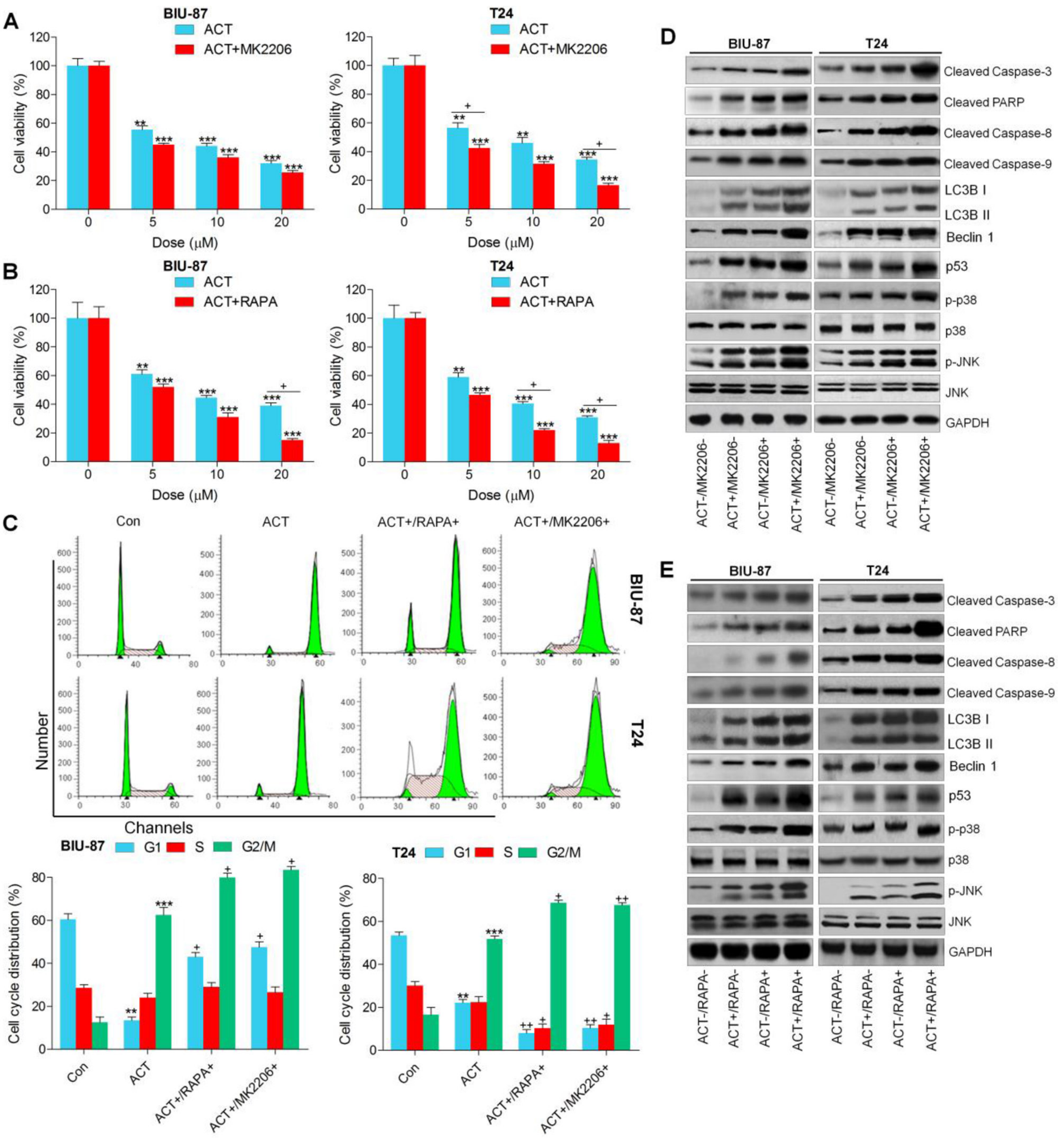

Supplementary Figure 2: AKT pathway is involved in cell cycle arrest, apoptosis and autophagy triggered by actein in human bladder cancer cells. (**A**) Bladder cancer cells were pre-treated with AKT inhibitor of MK2206 (1 mM) for 2 h, followed by ACT treatment at various concentrations for 24 h. And MTT analysis was conducted to evaluate the cell viability. $^{+}p < 0.05$, $^{++}p < 0.01$ and $^{+++}p < 0.001$. (**B**) BIU-87 and T24 cells were pre-cultured with mTOR inhibitor, Rapamycin (RAPA, 1 mM) for 2 h, and then ACT was treated to cells for another 24 h. Subsequently, MTT analysis was used to calculate the cell viability. (**C**) BIU-87 and T24 cells were treated as indicated, and then flow cytometry analysis was conducted to evaluate the cell cycle arrest. The cell cycle distribution was then quantified. $^{+}p < 0.05$, $^{++}p < 0.01$ and $^{+++}p < 0.001$ versus the ACT group. (**D**, **E**) BIU-87 and T24 cells were pre-treated with MK2206 or RAPA for 2 h, followed by ACT treatment or not for further 24 h. Then, all cells were harvested for western blot assays. Data are represented as mean ± S.E.M. $^{*}p < 0.05$, $^{**}p < 0.01$, $^{***}p < 0.001$ versus the untreated group.
